# A Forty-Eight Hours' Week

**Published:** 1920-05-15

**Authors:** 


### A FORTY-EIGHT HOURS' WEEK.

THE Dublin Corporation, which makes fairly substantial grants annually to city hospitals, has announced that these will be continued only to institutions which recognise a forty-eight hours' week for nurses. There have been such altogether unparalleled doings in Dublin recently that the citizens may be said to have got beyond the surprise condition of mind. Otherwise the announcement would have been in the nature of a bomb. The Irish nurse is even harder worked than her English sister, and worse paid. Hence her feelings at the suggestion of such a revolution may be more easily imagined than described. Nevertheless she is level-headed. It has been advertised far and wide that Dublin hospitals are £100,000 in debt, and, although the Corporation grants are not to be despised, their continuance or their forfeiture are only minor factors where so startling a change is contemplated.

Coincident with the Corporation's threat the Nurses' Union, which is a trade union, has taken the matter in hand. A meeting to which all persons interested in nursing were summoned was held on Friday, April 30, at Denmark House, the headquarters of the union, to discuss the forty-eight hours' question, with special reference to private nursing. I was present, and, if I may be permitted, I should like to congratulate Miss Bennett, who presided, and all those who took part in the proceedings on the reasonableness and high tone of their deliberations. Miss Matheson, secretary to the College of Nursing in Ireland, contributed greatly to the success of the meeting by her practical suggestions, and so likewise did Mr. Nehrewes, secretary to the Temperance Male Nurses' Corporation.

Everybody, including the chairman, Miss Bennett, appeared to be impressed with a sense of the delicate and difficult issues which are involved, and at the same time the "atmosphere" was throughout sympathetic with the nurse. Miss Bennett read a letter which she had received from a matron who strongly opposes the forty-eight hours' suggestion. It was particularly pathetic. This good lady expressed her complete willingness to spend herself in the cause, but in conclusion she admitted that she had one only anxiety—to be assured of a competence when she was spent!

I do not know the name of the writer. It was a private letter, not written for the limelight. Wherein lies its value. It conveyed in every line devotion even unto death. No shortening of the hours of duty, but just a pang lest age or infirmity might find her in the almshouse. Compare this with the sordid spirit of the age. More, and still more, and always more. Verily, such as she shall have their reward.

But what I would inquire is this—Are British men and women prepared to accept such sacrifice? Is it their will and wish that when they are well they shall thrive and grow rich, and, when they are sick, angels and martyrs shall minister to their wants for ten or twelve hours per diem, and then pine out the residue of their days in poverty and misery? This is the sort of sentiment that might be expected from South Sea Islanders, and the last that ought to issue from Christianity and proud civilisation. I have heard people talking quite contentedly about the nurse going to the almshouse, and also about "the spiritual side of nursing," and to me it is pure and unadulterated cant. I do not know whether the Dublin Corporation's scheme is possible just now and immediately. Probably not. But of this I am assured. It is the first big public declaration in favour of reasonable treatment for the nurses of the country.

Miss Matheson pointed out that it would be unreasonable, not to say impossible, to expect any nurse to stop short at the end of eight hours' work, especially in the case of a private nurse. She suggested, however, that it might be made up to her by allowing time off after a critical stage had been passed. A ninety-six-hour fortnight, for example, might meet the difficulty. Some speakers of the trade-union school suggested a higher rate of pay for overtime. But the more experienced, and, if I may say so, the more enlightened, pointed out that the aim was not gain, but reasonable working hours.

Concerning one point of major importance there seemed to be complete unanimity. Miss Bennett stated that, concerning the Bill now before Parliament establishing a general eight hours' day, English private nurses desired to be able to agree or to disagree, at their pleasure. Obviously, this would leave the situation worse than it is at present. It would establish a system of open competition, and pave the way for the entry of a vast army of untrained and falsely called nurses.

The end of the whole matter was that the meeting at Denmark House decided that, although extremely useful for its purpose, it was too small and too unrepresentative to arrive at any authoritative conclusion. A larger meeting is therefore to be called in some such building as the Mansion House, where the subject can be thrashed out by a larger gathering, without bias and on independent ground. The impression of the meeting left on my mind is that Irish nursing interests are tending towards unity. The faction element is disappearing, and nurses are beginning to realise that they are members of a great national and international profession, and that their interests are common.

IERNE.

